# Diagnostic Complexity of Recurrent Ascites: Pancreatic Ascites Mimicking Portal Hypertension

**DOI:** 10.7759/cureus.101751

**Published:** 2026-01-17

**Authors:** Idan Grossmann, Harshavardhan Sanekommu, Sidra Ahsan, Evgeniya Angelova, Lee Peng

**Affiliations:** 1 Department of Internal Medicine, Hackensack Meridian Health Jersey Shore University Medical Center, Neptune, USA; 2 Department of Gastroenterology and Hepatology, Hackensack Meridian Health Jersey Shore University Medical Center, Neptune, USA; 3 Department of Pathology, Hackensack Meridian Health Jersey Shore University Medical Center, Neptune, USA

**Keywords:** endoscopic ultrasound (eus), necrotizing pancreatitis complication, pancreatic ascites, portal hypertension, recurrent ascites

## Abstract

Recurrent ascites is a common medical condition that can arise from various underlying causes. Although it is frequently associated with cirrhosis, recurrent ascites can have multisystemic and multifactorial etiologies. Pancreatic ascites is a relatively uncommon cause of ascites. We present the case of a 68-year-old man with a history of alcohol use disorder and necrotizing pancreatitis, which required multiple interventions. The patient developed recurrent ascites, necessitating repeated paracentesis. Initially, the workup suggested that the ascites was due to portal hypertension secondary to cirrhosis. However, a high index of suspicion prompted further investigation. Given the improvement in both pancreatitis and ascites over the following weeks, the dominant etiology was determined to be pancreatic ascites. This case highlights the importance of considering a broad differential diagnosis when evaluating recurrent ascites, particularly when the etiology is not immediately clear.

## Introduction

Recurrent ascites is characterized by repeated accumulation of fluid in the peritoneal cavity, often requiring multiple paracentesis procedures. The most common cause of recurrent ascites is advanced liver cirrhosis with portal hypertension. Other etiologies also include malignancy, heart failure, renal disease, and, less commonly, pancreatic disease, which is an uncommon reason [1,2].

Pancreatic ascites is a rare complication, occurring in about 3% of patients with pancreatitis [3]. However, the prevalence of pancreatic ascites is increased in patients with complicated pancreatitis, and it correlates with the disease severity [4].

We present a patient with a history of alcohol use disorder who developed acute pancreatitis complicated by necrotizing pancreatitis, accompanied by recurrent ascites. Given the patient’s alcohol history and the common association between ascites and cirrhosis, we initially suspected the ascites to be secondary to portal hypertension from cirrhosis. However, further evaluation ruled it out, and as both the pancreatitis and ascites improved, the predominant cause was determined to be pancreatic ascites. This case highlights the diagnostic complexity of recurrent ascites in patients with multiple potential causes and emphasizes the importance of considering all possibilities.

## Case presentation

A 68-year-old man with a past medical history of hypertension, hyperlipidemia, alcohol use disorder, and an episode of acute pancreatitis complicated by necrotizing pancreatitis requiring prior endoscopic necrosectomies with stent placement presented to the hospital with complaints of worsening abdominal pain and distention, associated with swelling of the legs. He had recently completed a prolonged two-month hospitalization during which he underwent several endoscopic necrosectomies and multiple therapeutic paracenteses for ascites and was discharged home with planned outpatient follow-up and an additional scheduled necrosectomy.

After discharge, he experienced progressive abdominal distention, reduced oral intake, and worsening abdominal pain, prompting his return to the hospital.

Physical examination demonstrated diffuse abdominal distention and tenderness, along with bilateral lower extremity edema. Laboratory testing revealed leukocytosis of 19,000/µL, sodium at 132 mmol/L, alkaline phosphatase at 208 U/L, and chronic mild elevations in aspartate aminotransferase (AST) at 72 U/L and alanine aminotransferase (ALT) at 106 U/L. Despite a high serum ascites albumin gradient (SAAG), the patient’s normal synthetic liver function raised suspicion for an atypical etiology (Table 1).

**Table 1:** Initial laboratory values on admission AST: Aspartate aminotransferase, ALT: alanine aminotransferase, INR: international normalized ratio.

Laboratory Parameter	Result	Reference Range
White blood cell count	19,000 /µL	4,000-11,000 /µL
Hemoglobin	14.2 g/dL	13.5-17.5 g/dL
Platelet count	210,000 /µL	150,000-400,000 /µL
Sodium	132 mmol/L	135-145 mmol/L
Alkaline phosphatase	208 U/L	40-129 U/L
AST	72 U/L	10-40 U/L
ALT	106 U/L	7-56 U/L
Total bilirubin	0.3 mg/dL	0.2-1.2 mg/dL
INR	1.31	0.8-1.2
Serum lipase	150 U/L	10-60 U/L

**Figure 1:**
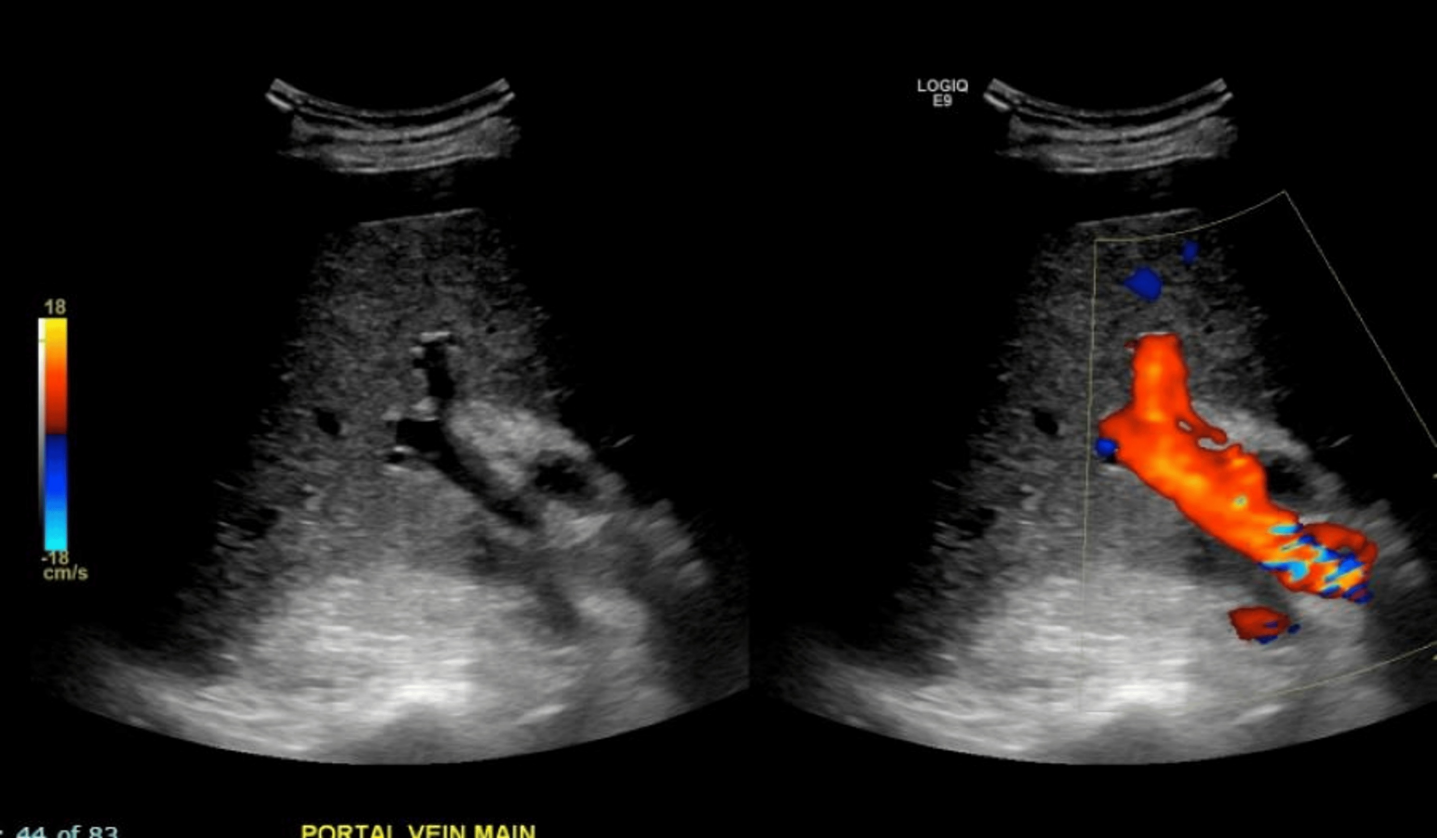
Right upper quadrant ultrasound demonstrating nodular liver contour, patent main portal vein, and absence of cholelithiasis or definite masses

**Figure 2:**
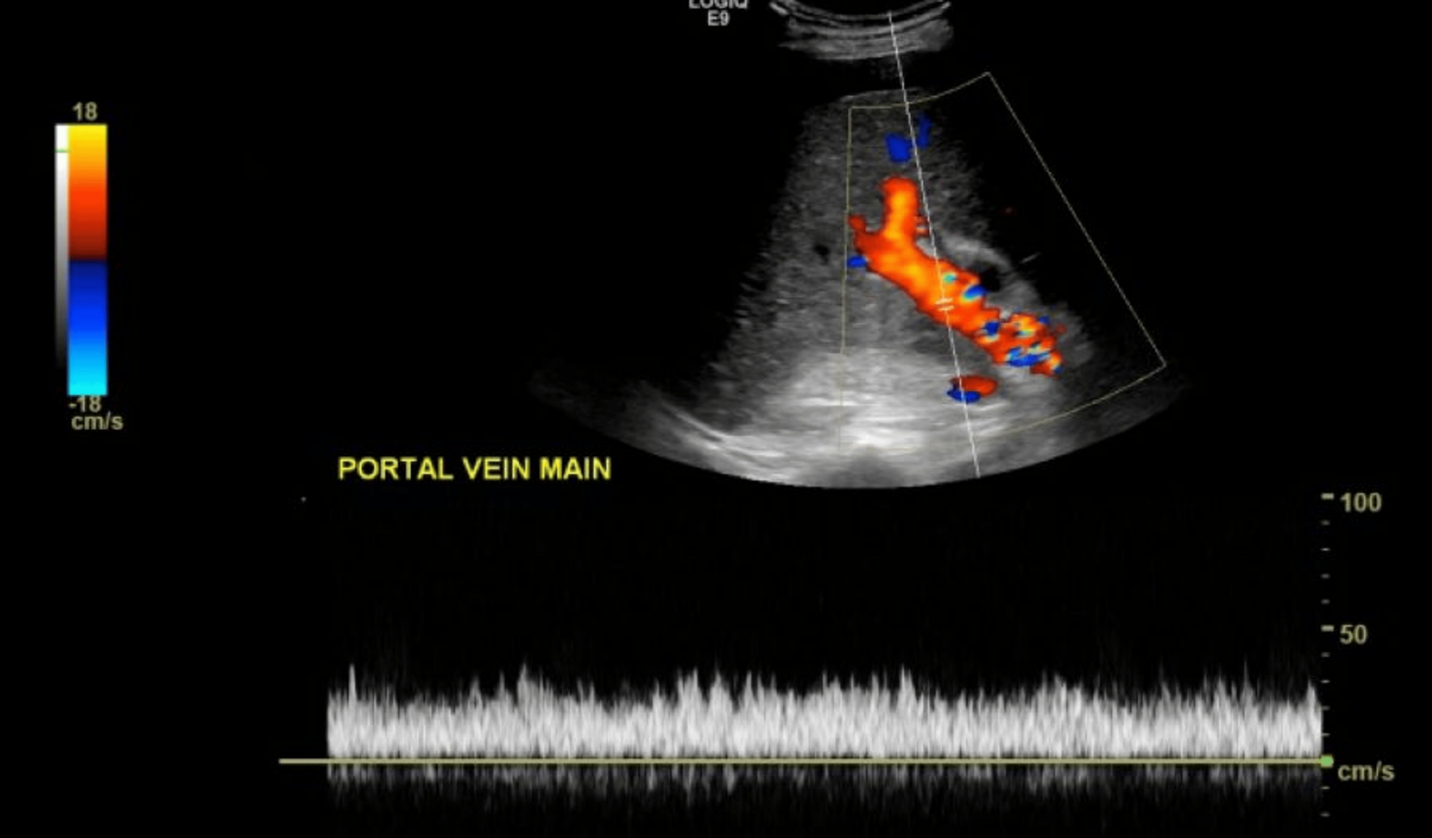
Doppler ultrasound of the main portal vein demonstrating patent vessel with normal hepatopetal flow

The right upper quadrant ultrasound with Doppler demonstrated ascites, a partially visualized heterogeneous pancreas with irregular fluid collection, a nodular liver contour, no cholelithiasis or definite masses, and a patent main portal vein (Figures 1, 2).

**Figure 3:**
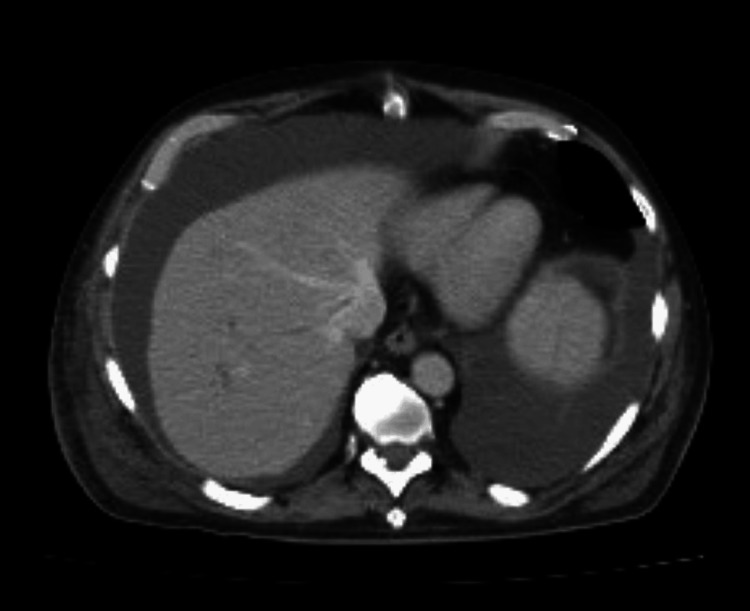
Axial CT of the abdomen and pelvis demonstrating large-volume ascites

**Figure 4:**
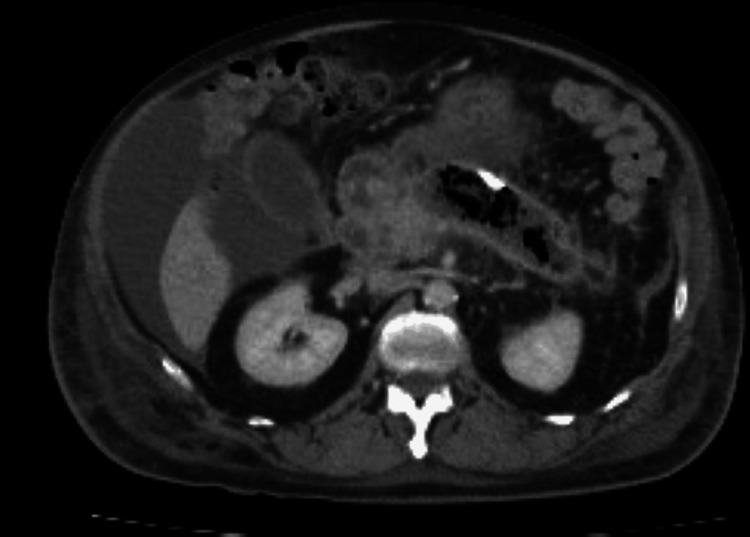
Axial CT of the abdomen showing persistent peripancreatic fluid collections

A CT of the abdomen and pelvis showed persistent peripancreatic fluid collections accompanied by ascites (Figures 3, 4).

**Figure 5:**
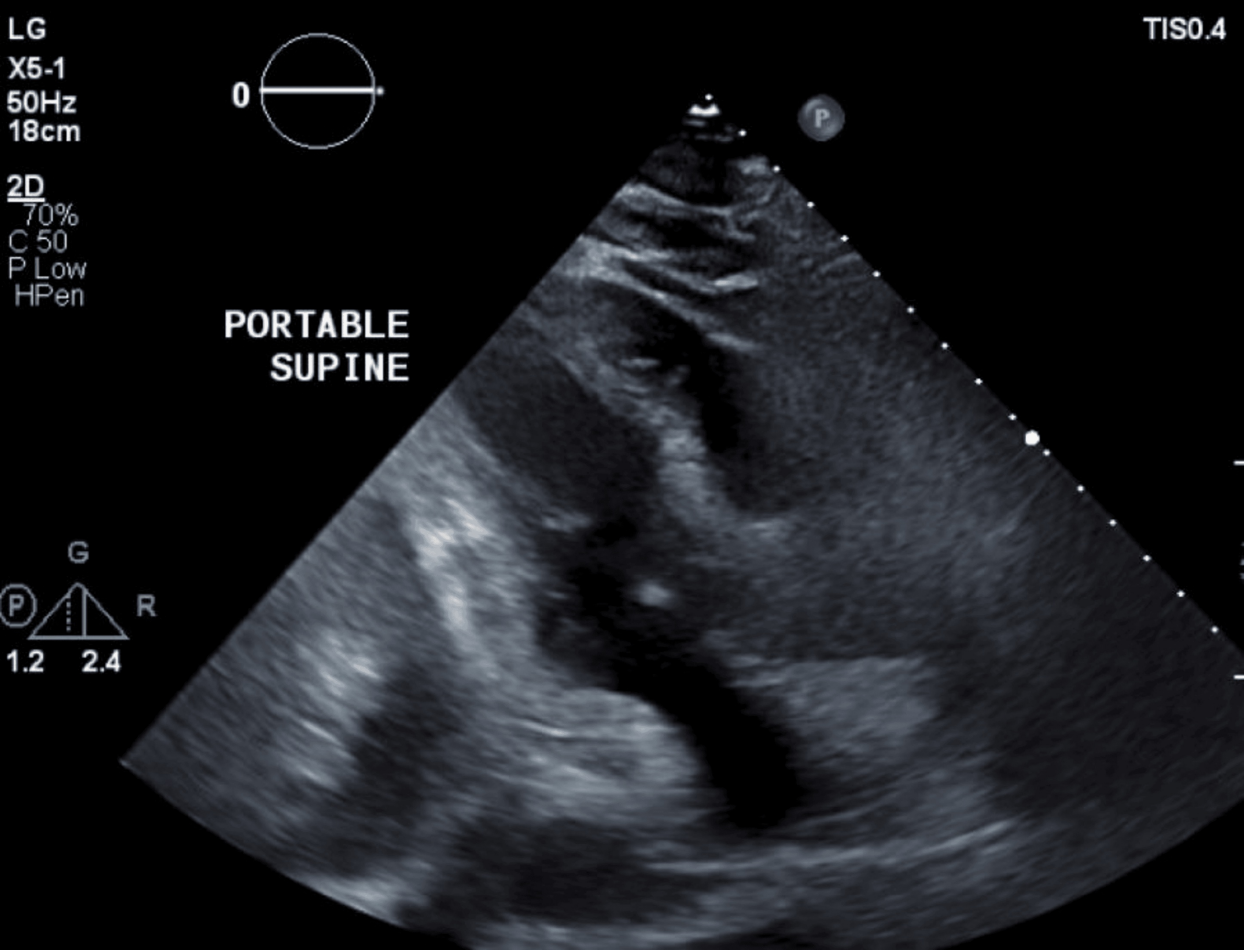
Transthoracic echocardiography showing normal left ventricular systolic function (ejection fraction (EF) 65%–70%) with mild (grade I) diastolic dysfunction

Echocardiography showed normal systolic function with an ejection fraction of 65%-70% and grade I (mild) diastolic dysfunction (Figure 5).

A first paracentesis was performed, resulting in the removal of 3.6 liters of ascitic fluid. Analysis of the fluid demonstrated an SAAG of 1.5 g/dL, consistent with portal hypertension. The fluid was clear and yellow in appearance, with a total protein of 1.2 g/dL, 59% segmented neutrophils, glucose of 95 mg/dL, lactate dehydrogenase (LDH) of 120 U/L, and an amylase level of 45 U/L. Cultures were negative for bacterial growth.

**Table 2:** Ascitic fluid analysis

Parameter	Result
Appearance	Clear, yellow
Serum ascites albumin gradient (SAAG)	1.5 g/dL
Total Protein	1.2 g/dL
White blood cells (WBC)/Neutrophils	59% segmented neutrophils
Glucose	95 mg/dL
Lactate dehydrogenase(LDH)	120 U/L
Amylase	45 U/L
Culture	Negative

**Figure 6:**
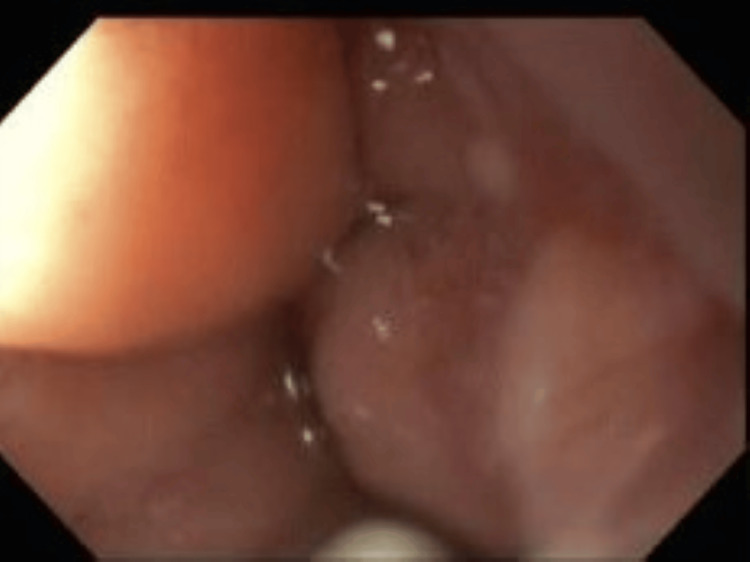
Endoscopic view demonstrating severe duodenal edema and congestion causing luminal narrowing

Despite the high SAAG, the patient’s normal synthetic liver function raised suspicion for an atypical etiology. Subsequently, endoscopic evaluation revealed severe duodenal edema and congestion, resulting in narrowing of the duodenal lumen and preventing safe endoscopic retrograde cholangiopancreatography (ERCP) (Figure 6). Previously placed stents were visualized and drained a large volume of purulent fluid. Approximately 80% of necrotic debris was removed and new stents were placed.

Despite appropriate management of pancreatic necrosis, the patient continued to develop large-volume recurrent ascites. A repeat necrosectomy was performed, removing additional necrotic debris and exchanging cystgastrostomy stents. Following a repeat necrosectomy, a second paracentesis removed 5.6 L of clear amber fluid, again showing high SAAG without significant amylase elevation.

Although the ascites initially appeared to be portal hypertensive, the patient had normal synthetic liver function and no prior history of ascites. Hepatology consultation was obtained due to concern for underlying cirrhosis; however, the patient lacked thrombocytopenia, had only mild cholestatic enzyme elevations, and imaging findings were not fully consistent with cirrhosis. To clarify the etiology, an upper endoscopic ultrasound (EUS) with portal pressure measurement was performed, demonstrating a normal portal pressure gradient of 7-8 mmHg.

**Figure 7:**
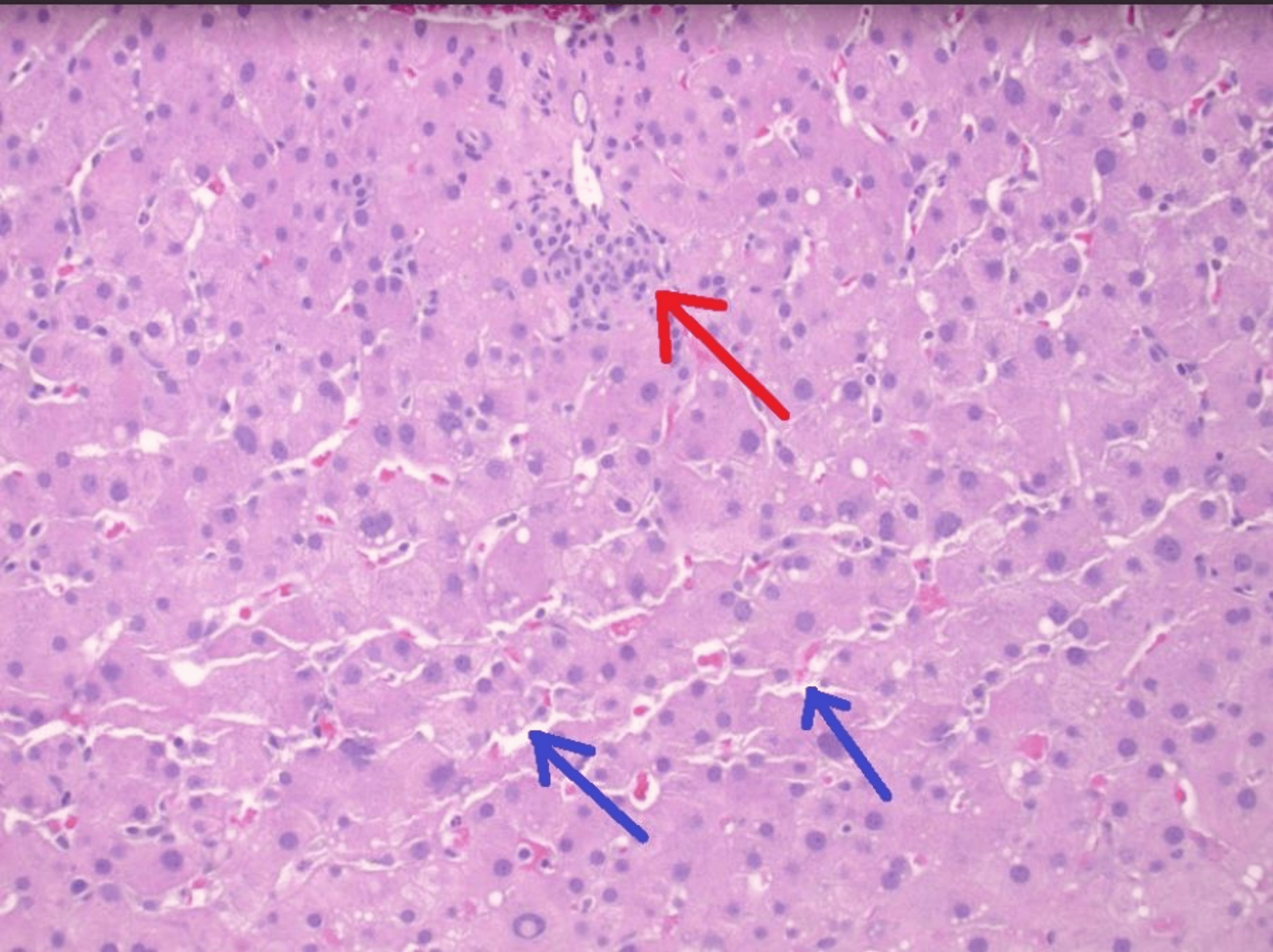
Portal tracts show minimal inflammatory changes (red arrow) without significant numbers of plasma cells or eosinophils. Slightly dilated sinusoids (blue arrows) throughout the parenchyma.

**Figure 8:**
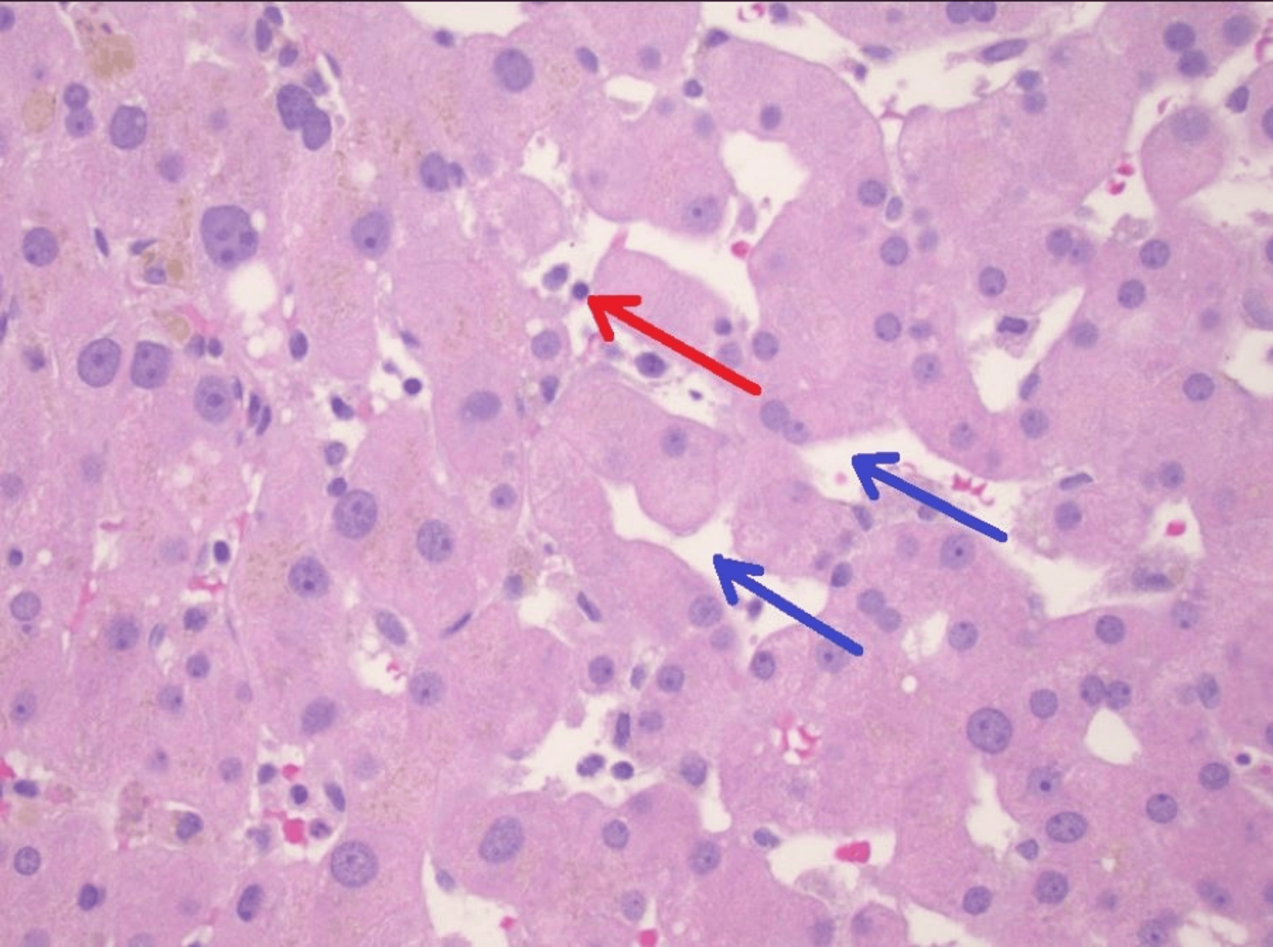
The hepatic lobules show minimal nonspecific inflammatory changes with rare intrasinusoidal lymphocytes (red arrow) as well as minimal sinusoidal dilatation (blue arrows).

**Figure 9:**
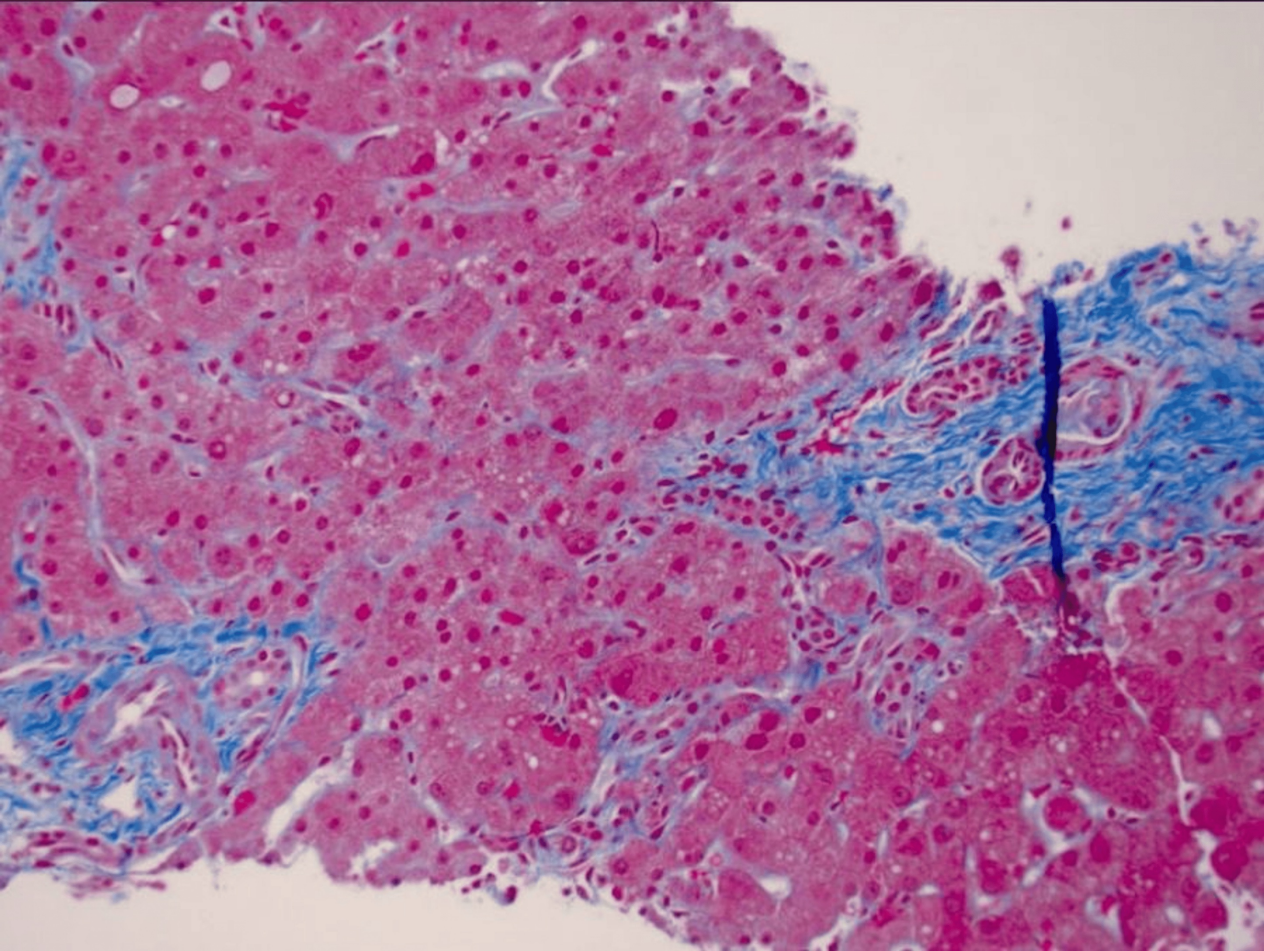
Mild portal/periportal fibrosis (stages 1-2 of 4).

**Figure 10:**
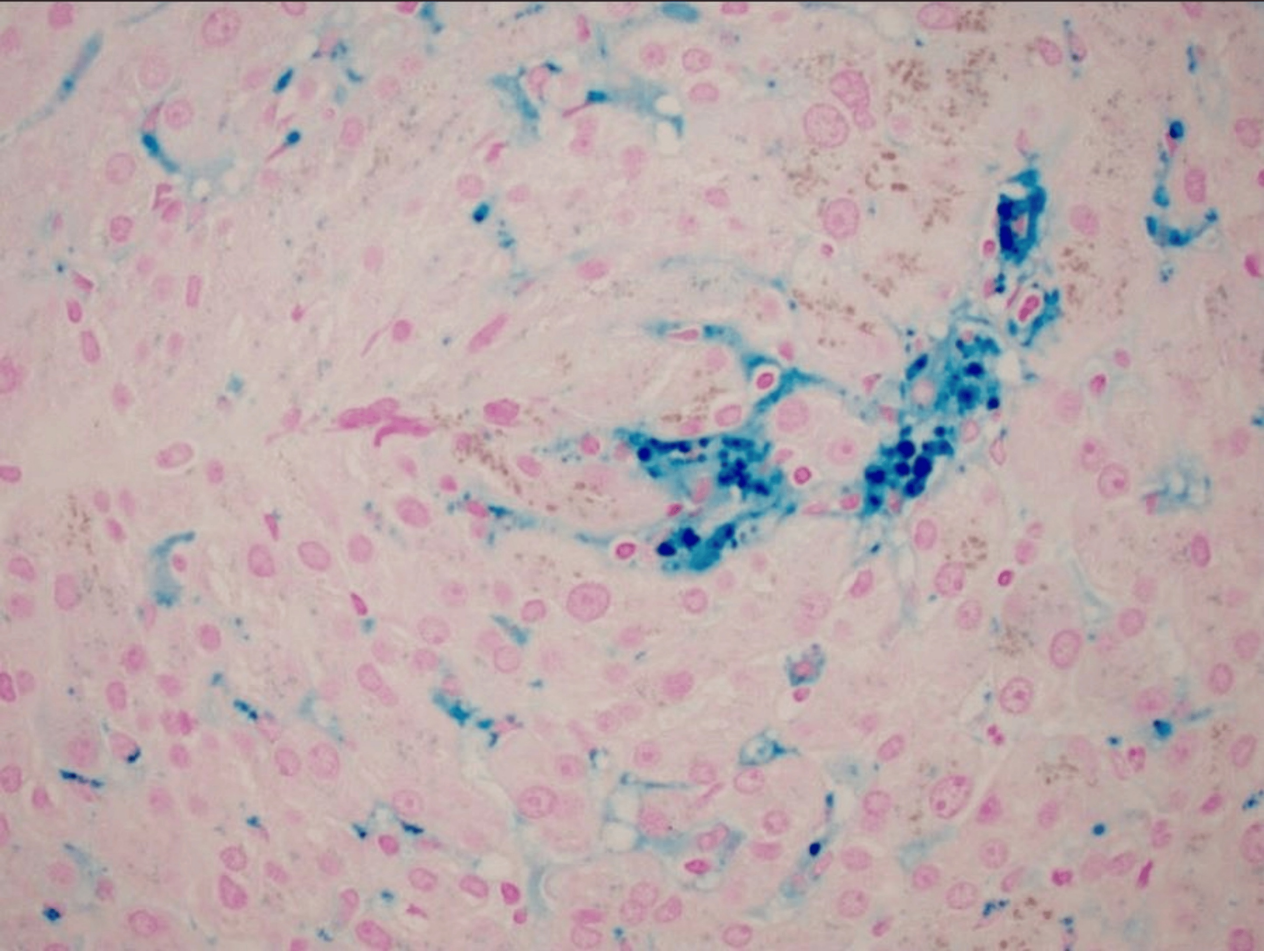
Iron deposition predominantly within Kupffer cells (secondary overload pattern).

EUS-guided liver biopsy demonstrated preserved liver architecture and in tact bile ducts and vasculature, without evidence of cirrhosis, autoimmune hepatitis, or steatohepatitis. Minimal nonspecific portal and lobular inflammation (Figures 7, 8). Minimal sinusoidal dilatation with associated sinusoidal fibrosis was noted (Figure 9) and moderate iron deposition predominantly within Kupffer cells (Figure 10).

Given the patient’s persistent recurrent ascites with a high SAAG, normal portal pressure measurements, and largely unremarkable liver histology, including only minimal sinusoidal dilatation and fibrosis, with no evidence of cirrhosis or other chronic liver disease, combined with only mild diastolic dysfunction and no signs of decompensated heart failure, both cardiac and hepatic causes were considered unlikely. The ascites was therefore ultimately attributed to pancreatic origin. During outpatient follow-up, both the patient’s pancreatic condition and ascites improved concurrently, further supporting pancreatic ascites as the primary etiology.

## Discussion

Ascites is a common presentation in the emergency department and can arise from multiple multisystemic causes, with portal hypertension secondary to cirrhosis being the most frequent [1,2]. Our case illustrates the complexity of determining the underlying etiology. The patient, with a significant history of alcohol use, initially presented with ascitic fluid analysis consistent with portal hypertension, suggesting cirrhosis as the cause. However, further invasive workup ruled it out. Liver biopsy showed signs of congestion, raising the possibility of a cardiac origin, but the patient’s mild diastolic dysfunction and absence of symptoms of decompensated heart failure made this unlikely. In contrast, the timing of ascites onset, which coincided with necrotizing pancreatitis, along with the resolution of ascites following improvement of pancreatitis, normal portal pressure measurements, and an unremarkable liver biopsy, supports pancreatic ascites as the most likely cause of this presentation.

Pancreatic ascites is a rare complication of pancreatitis, with an estimated incidence of about 1%-5% among patients with acute pancreatitis and higher rates in those with complications such as necrotizing pancreatitis [3]. These patients are more likely to develop recurrent or persistent ascites due to ongoing leakage of pancreatic fluid, and the presence of ascites correlates with the disease severity [5].

Although pancreatic ascites is classically characterized by elevated amylase in the ascitic fluid, amylase-negative or mildly elevated amylase has been reported, as in our case, where the patient had only mildly elevated ascitic amylase. A prospective study found that ascitic fluid amylase is not reliably elevated in acute pancreatitis - 71.9% of patients with ascites had low amylase levels despite having a low SAAG (87.8%) consistent with a pancreatic etiology. The study demonstrated that only 28.1% of acute pancreatitis patients with ascites had high ascitic amylase levels, meaning the majority presented with low or normal levels despite clear clinical and imaging evidence of pancreatic disease [6]. High ascitic amylase (>1000 U/L) is specifically associated with duct disruption, which represents a particular subset of pancreatic ascites rather than all cases occurring in acute pancreatitis. Therefore, markedly elevated ascitic amylase should raise suspicion of pancreatic duct injury rather than assuming a universal finding in acute pancreatitis-related ascites [7]. In addition, ascitic fluid amylase is found to have limited prognostic value in acute pancreatitis, while other markers such as LDH levels are more clinically relevant markers for predicting complications and mortality [8-10].

Similarly, while pancreatic ascites is traditionally described as having a low SAAG and high protein content with elevated pancreatic enzymes, these features are not uniformly present in acute or complicated pancreatitis. Ascites related to severe pancreatic inflammation may deviate from classic biochemical profiles and, in some cases, mimic portal hypertensive ascites, particularly when ductal disruption is not demonstrable [7]. In our patient, the ascitic fluid had a high SAAG of 1.5 g/dL, total protein of 1.2 g/dL, amylase of 45 U/L, and LDH of 120 U/L, while serum lipase was mildly elevated at 150 U/L. These values reflect an atypical presentation, emphasizing that pancreatic ascites can occur with only mild enzyme elevations and may mimic portal hypertensive ascites, highlighting the need for integration of clinical history, imaging, and exclusion of hepatic or cardiac causes in establishing the diagnosis.

This case demonstrates the wide variety of potential underlying causes of ascites, which can make identifying the primary etiology particularly challenging in patients with multiple contributing factors. It also highlights the importance of a systematic and comprehensive evaluation, including clinical history, imaging, laboratory studies, and, when necessary, invasive testing, to accurately determine the source and guide appropriate management.

## Conclusions

Pancreatic ascites is a rare but important cause of recurrent ascites that can mimic portal hypertension, even when ascitic fluid analysis suggests a high SAAG and ascitic amylase is not markedly elevated. Accurate diagnosis requires a comprehensive evaluation, including clinical history, imaging, invasive testing such as portal pressure measurement, and sometimes liver biopsy, to rule out hepatic or cardiac causes. Early recognition and appropriate management of pancreatic ascites can prevent unnecessary interventions and guide targeted therapy.
